# Faith in the Midst of Trials: Understanding Depression and Christian Coping Strategies Among Cancer Patients in Eastern Europe

**DOI:** 10.7759/cureus.84484

**Published:** 2025-05-20

**Authors:** Daniel Jugrin, Ana Anca Jitaru, Crischentian Brinza, Alexandru Burlacu

**Affiliations:** 1 Center for Interreligious and Intercultural Studies and Dialogue, University of Bucharest, Bucharest, ROU; 2 Cardiology, Faculty of Medicine, University of Medicine and Pharmacy "Grigore T Popa", Iași, ROU

**Keywords:** cancer patients, christian orthodox faith, coping strategies, depression, mental health, religious coping

## Abstract

Depression is notably prevalent among patients with neoplastic diseases, significantly impacting their quality of life and coping mechanisms. Effective coping strategies are crucial for better adaptation, enhanced quality of life, and potentially longer survival in cancer patients. This study explores the role of religion and spirituality, particularly from a Christian Orthodox perspective, in coping with cancer-related challenges. The study employed a qualitative approach, analyzing the existing literature on psychosocial oncology and religious coping strategies. It focused on the cognitive and behavioral aspects of religious coping mechanisms, particularly within the Christian Orthodox context in Romania. Data from various studies, including surveys and patient interviews, were reviewed to understand how religious beliefs and practices influence coping in cancer patients. The findings reveal that a collaborative relationship with God, characterized by shared control over the situation, positively influences patients' belief in their ability to cope with cancer. In contrast, a passive coping style is associated with a diminished sense of personal coping competence. Many patients reported relying on prayer and faith, finding these to be effective in managing the stress of their condition. Religious and spiritual resources are significant in coping with cancer, especially in facing life-threatening situations. From the Christian Orthodox viewpoint, disease is seen as an opportunity for spiritual growth and reconciliation, which aids in the coping process. Integrating these spiritual dimensions into care models can enhance support for cancer patients.

## Introduction and background

Depression, characterized by persistent sadness and a lack of interest or pleasure in previously rewarding or enjoyable activities, is one of the most common mental health challenges worldwide. As of 2012, an estimated 350 million people globally have experienced depression, underscoring its status as a major public health concern [1]. The lifetime risk of developing major depressive disorder is notably gender-dependent, with rates of 5%-12% for men and 10%-25% for women, while the prevalence of depressive symptoms stands at 2%-3% for men and 5%-12% for women at some point in their lives [2]. These statistics highlight the widespread nature of this condition, which transcends geographical and cultural boundaries.

In 1998, the World Health Organization predicted that by 2020, depression would rank as the second most debilitating disease globally, just behind heart disease. This projection draws attention to the significant impact of depression not only on individual well-being but also on societal and economic structures. Ernst Berndt and colleagues from the Massachusetts Institute of Technology quantified this impact in the United States, estimating the economic burden of depression to be around $44 billion, a figure comparable to the costs associated with coronary heart disease [3]. This economic analysis reveals the substantial financial implications of depression, driven by factors such as healthcare expenditures, lost productivity, and the broader societal costs. In the predominantly Christian Orthodox regions of Eastern Europe, such as Romania, religious beliefs and spiritual traditions play a significant role in shaping psychological responses to illness, including cancer.

Specifically, in the realm of chronic illnesses like cancer, depression takes on an added dimension of complexity. Cancer patients are particularly vulnerable to depression, which can arise as a direct response to their illness, treatment side effects, or the psychological burden of a cancer diagnosis [4]. Studies have shown that depression can significantly affect cancer patients' quality of life, treatment adherence, and overall prognosis [4,5]. However, the intersection of depression and cancer is not just a medical issue; it is deeply intertwined with patients' psychological and spiritual coping mechanisms [5].

Spirituality is increasingly recognized as a meaningful psychological resource in the cancer trajectory. A review published in 2021 systematically examined the relationship between spirituality and emotional well-being in oncology patients [6]. The most cross-sectional studies included in the analysis reported a positive association between spiritual orientation and psychological well-being [6]. In a study of advanced cancer patients attending a palliative care clinic, the authors reported that nearly all the participants identified as both spiritual and religious [7]. Despite this, spiritual pain was present in approximately 44% of the patients and was significantly associated with poorer spiritual quality of life and lower perceived religiosity. Notably, individuals reporting spiritual pain also exhibited more severe physical and emotional symptoms, including increased levels of anxiety and depression. These findings highlighted the relevance of spirituality as a coping dimension and reinforce the importance of addressing these concerns in comprehensive cancer care [7].

Christian Orthodox traditions, particularly in Eastern Europe, with their rich spiritual heritage, offer unique perspectives and practices for coping with depression, particularly in the context of life-threatening illnesses like cancer. These practices, which may include prayer, meditation, participation in religious rituals, and community support, have been observed to provide significant psychological comfort and resilience. [8]. While this study centers on Christian Orthodox coping strategies, it is worth noting that religious coping also plays a significant role in other traditions, including Islamic, Buddhist, and secular humanist frameworks. However, the mechanisms and clinical integration of such strategies often differ, underscoring the need for culturally specific analysis [9].

However, there is a notable gap in the scientific literature regarding the efficacy and mechanisms of these Christian Orthodox coping strategies. Therefore, we aimed to address this gap by exploring how Christian Orthodox coping strategies can aid in managing depression among cancer patients. By integrating psychological, medical, and spiritual perspectives, this study aims to contribute to a more holistic understanding of depression management in the context of chronic illness. The ultimate goal is to offer insights that could inform patient care practices, enriching the toolkit of healthcare professionals and caregivers in supporting cancer patients battling depression.

## Review

Materials and methods

We employed a qualitative research design to investigate the intricate dynamics of religious coping strategies among cancer patients within the Christian Orthodox paradigm, specifically concentrating on the Eastern European context, with a focal point on Romania. In order to comprehensively retrieve relevant literature, we conducted a search across various databases, including PubMed, Embase, and Scopus (Supplementary file, Search results). The search strategy ensured the retrieval of eligible studies within the intersection of psychosocial oncology, depression in cancer patients, and religious coping mechanisms. The main keywords used in the search included: “cancer”, “depression”, “religious coping”, “Christian Orthodox coping strategies”, “spirituality”, “psychosocial oncology”, and “Eastern European cancer patients”.

This eligibility assessment aimed to select studies specifically addressing the psychosocial dimensions of cancer, exploring depression in cancer patients, and investigating the utilization of religious coping strategies, with a particular emphasis on the Eastern European and Christian Orthodox context. Studies were included if they met the following criteria: (1) the population consisted of cancer patients of any age or cancer type; (2) the study addressed aspects of depression, psychological distress, or emotional well-being in the context of cancer; (3) the study discussed religious or spiritual coping mechanisms, with particular relevance to the Christian Orthodox tradition or Eastern European context. Studies were excluded if they (1) did not focus on cancer patients, (2) addressed religiosity or spirituality without linking them to coping or psychological outcomes, (3) were limited to theoretical or theological commentary without empirical data, or (4) were duplicates or without full-text availability. Discrepancies in the eligibility assessment were resolved through consensus.

The present review explored a spectrum of outcomes to unravel the interplay between religious coping strategies and the psychological well-being of cancer patients within the Christian Orthodox context in Eastern Europe. The main outcomes encompassed evaluating the effectiveness of religious coping in alleviating depressive symptoms, enhancing adaptive mechanisms, and fostering an improved quality of life for individuals facing life-threatening illnesses. Furthermore, we have also explored the behavioral aspects of religious coping, assessing the impact of practices such as prayer, participation in religious rituals, and engagement with spiritual communities on patients' coping mechanisms.

Results

**Figure 1:**
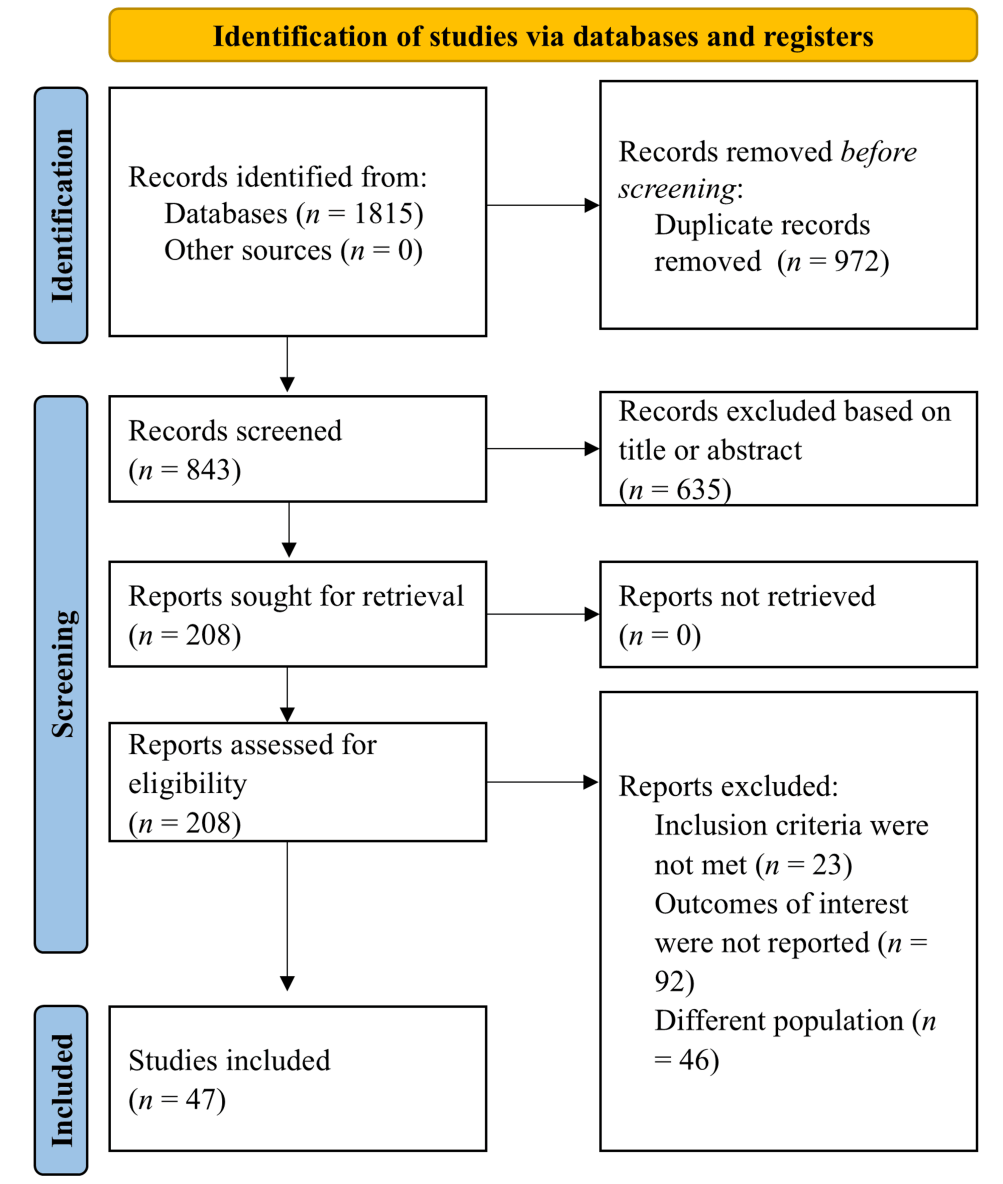
Flow diagram of selecting studies in present analysis.

A total of 1,815 records were identified through electronic database searching. After the removal of duplicates, 843 titles and abstracts were screened. Ultimately, 47 studies were included in the narrative synthesis, as outlined in the Preferred Reporting Items for Systematic Reviews and Meta-Analyses (PRISMA) flow diagram (Figure 1).

1. Depression in Cancer Patients

Depression ranks as the most prevalent psychological symptom in patients with neoplastic diseases, exhibiting a considerable presence with rates estimated at 38% for major depression and 58% for a broader spectrum of depressive syndromes [10,11]. Its prevalence is notably higher in the context of cancer, where it not only emerges as a frequent subject in studies linking psychological factors with cancer progression and mortality but also distinguishes itself as a more common and persistent psychological issue in cancer patients compared to the general population [12].

However, the term 'depression' encompasses a wide and somewhat nebulous range of conditions. This spectrum extends from mild, transient mood fluctuations, which are a universal human experience, to severe psychiatric disorders that inflict profound distress and can potentially escalate to suicidal ideation [13]. The current classification and terminology surrounding depressive states present significant complexities [1]. Particularly in the context of oncology, a nuanced approach to categorizing depressive states is imperative. This can be effectively achieved by differentiating between 'depressive reactions' and 'depressive diseases'. Depressive reactions entail a transient mood deterioration, often triggered by psychological stress, with the severe form typically not exceeding a duration of one week. In contrast, depressive diseases, or clinical depression, may arise independently of external stressors. They are characterized by symptoms that not only differ qualitatively but are also generally more severe and prolonged than the intense yet fleeting feelings of sadness that define depressive reactions [14].

*Psychological determinants*: In the majority of cases involving depressive diseases, a significant stressful life event can be identified as a precipitating factor. This aspect is particularly salient in cancer patients, who often endure multifaceted stressors. These include the psychological impact of receiving a cancer diagnosis, undergoing potentially mutilating surgeries, and enduring the side effects of radiotherapy and chemotherapy, ranging from loss of mobility and independence to chronic pain and the anticipation of mortality [15]. Furthermore, the risk of depression escalates in individuals lacking robust support systems, encompassing stable personal relationships, fulfilling employment, financial stability, and satisfactory living conditions.

*Biophysical contributors*: In cancer patients, metabolic dysfunctions can arise either as a direct consequence of the neoplastic process or as a side effect of anticancer treatments, both of which can precipitate mood alterations. Notably, severe depressive episodes in this population are frequently correlated with hormonal imbalances and electrolyte metabolism disturbances. When these physiological changes are induced by the disease or its treatment, a condition termed secondary (or derivative) depression may emerge. Intriguingly, there is evidence suggesting a disproportionately high occurrence of depressive symptoms preceding the physical manifestations of cancer. This phenomenon may be attributable to certain neoplasms secreting mood-altering substances or to a hypothesis that pre-existing depressive states could diminish physical resistance to oncological diseases [14].

*Psychiatric manifestations*: The mental symptoms observable in cancer patients are diverse and multifaceted. These include a pervasive depressed mood; variable patterns of crying (ranging from excessive crying at minor provocations to an expressed desire to cry without the ability to do so); a marked loss of interest or pleasure in previously enjoyable activities; concentration difficulties; feelings of excessive guilt, worthlessness, or perceived burdensomeness; profound pessimism and hopelessness regarding the future, often accompanied by suicidal ideation; heightened anxiety and irritability; significant distress; and spiritual suffering.

*Physical manifestations*: Among cancer patients, physical symptoms commonly associated with depression include sleep disturbances, nausea, diminished appetite leading to weight loss, pervasive loss of energy, constant fatigue, and various forms of pain, such as headaches or localized pain in different body regions [14,16]. These symptoms, while not exclusive to depression, often exacerbate the psychological burden of the illness.

*Epidemiological variability*: The prevalence of depression as a comorbid condition with cancer, and as a consequence of cancer treatment, demonstrates significant variability based on the cancer's location. For instance, depression rates in patients with head or neck cancers are notably high, ranging from 25% to 52%. In contrast, cancers of the pancreas, liver, colon, lung, brain, bladder/kidney, and prostate are associated with depression rates between 7% and 9.7%. Among breast cancer survivors, the prevalence of depression spans a wide range, from 1.5% to 46% [17]. These disparities underscore the need for cancer-specific psychological assessments and interventions.

*Suicide rates as an indicator*: A critical observation is that instances of extremely severe depression are relatively infrequent among cancer patients, a conclusion primarily drawn from studies of their suicide rates [18]. This finding suggests that while depression is common, its most extreme manifestations might be less prevalent in this population.

*Psychological impact by cancer type*: Specific cancer types display distinct patterns regarding the prevalence and onset of depression. Lung cancer patients, for example, often exhibit high depression rates even before their diagnosis is confirmed [19]. This contrasts with patients who have operable breast cancer, where depression is rare in the pre-diagnosis stage. Anxiety, whether accompanied by depression or not, is a common experience among patients undergoing diagnostic tests for suspected cancer [20]. Delays in hospital appointments or obtaining test results can exacerbate these psychological stresses. Upon receiving a cancer diagnosis, reactions vary significantly; some patients report a sense of relief at having clarity about their condition, while others may experience onset or worsening of depressive symptoms. Studies focusing on lung cancer patients indicate that depression is more prevalent in those who are not undergoing active cancer treatment compared to those receiving radiotherapy and palliative chemotherapy, despite the latter's adverse side effects [21].

2. Religious Coping with Cancer

*Therapeutic outcomes in depressive cancer patients*: Effective treatment of depression in cancer patients has been shown to yield significant improvements across various domains. These enhancements include better sleep quality, increased appetite, improved concentration, heightened interest and joy in daily activities, and even augmented tolerance to oncological treatments [22]. Recent research further substantiates the notion that cancer patients who demonstrate a more effective coping mechanism are likely to experience better adaptation to their condition, enjoy a higher quality of life, and potentially have an extended survival rate [23].

*Conceptualizing coping*: Folkman [24] articulates coping as 'those ever-changing cognitive and behavioral efforts to manage specific external and/or internal demands that are assessed as demanding or exceeding a person's resources.’ This definition encapsulates coping as a dynamic process, encompassing both cognitive and behavioral strategies employed to navigate challenges that are perceived as taxing or surpassing one's capacities.

*Role of coping in cancer*: In the context of cancer, coping embodies a constellation of behaviors and cognitive activities initiated in response to distressing events, such as receiving a cancer diagnosis or grappling with a daunting prognosis. These response behaviors, which often emerge subconsciously, represent the patient's psychological endeavor to counteract the stressor. They aim to mitigate the disruptions caused by the diagnosis and the initiation of treatment, or to cope with the realization of facing non-curative, palliative care options [25]. These psychological responses are critical in preserving the patient's quality of life and maintaining a sense of control and resilience during their cancer journey.

*Research trends in coping with cancer*: Since 1985, approximately 30% of studies focusing on coping mechanisms have delved into the specifics of coping with cancer. Despite this substantial interest, the role of religion and spirituality as coping mechanisms in oncology has not been extensively explored. Remarkably, until 1998, a mere 1% of coping-related studies had investigated the utilization of belief systems in the coping process [26]. This oversight is notable considering the significant impact that religious and spiritual beliefs can have, both in cognitive terms (such as interpreting the disease as part of a divine plan) and in behavioral terms (like engaging in prayer or attending religious services).

*Definition of spiritual coping*: In light of these considerations, religious or spiritual coping can be conceptualized as the employment of cognitive and behavioral strategies, rooted in an individual's religious or spiritual beliefs, to confront and manage stressful life events [27].

*Emerging recognition in psychosocial oncology*: Recent advancements in psychosocial oncology have begun to acknowledge the spiritual responses that individuals with cancer often exhibit [28]. This acknowledgment is corroborated by findings indicating that many cancer patients refer to their religious beliefs when discussing their strategies for coping with highly stressful situations [29,30].

*The role of religion in cancer coping*: The onset of cancer often precipitates a profound disruption in the patient's sense of meaning/significance, perceived control, and self-esteem. Patients are frequently compelled to confront existential questions such as 'Why me?' and 'Why cancer?'. As control over their lives is transferred to medical professionals, feelings of helplessness ensue, and self-esteem can be adversely impacted. In these circumstances, religion can offer an alternative avenue for coping, mediated through two primary channels: the individual's own spiritual orientation and the support provided by religious representatives or clergy [31].

*Correlation between religiosity and depression remission*: Koenig et al. [32] conducted a seminal study revealing that high levels of intrinsic religiosity, defined as the deep internalization of faith as a primary motivator in individuals' lives [33], were predictive of a more rapid remission of major depressive disorder among elderly hospitalized patients. This correlation extends to cancer patients, where intrinsic religiosity has been positively associated with an increased sense of hope, meaning, and peace. Conversely, it shows a negative association with depressive symptoms and other adverse mood states [34].

*Integration of religious/spiritual resources in coping*: The incorporation of religious and spiritual dimensions in coping strategies has been a focal point for researchers exploring responses to stressful life events, particularly in the work of Pargament [29]. He emphasized how integrating religious resources, such as appraisals, beliefs, and coping behaviors, can enrich the existing coping patterns [24]. These religious and spiritual resources become increasingly pertinent in contexts of severe stress, such as life-threatening situations or the loss of life, typical in incurable diseases. Recent studies, including those by Halstead and Fernsler [35], underscore this significance. Their research, involving spontaneous letters from men and women, highlighted that prayer and trust in God, were frequently cited as the most commonly employed and effective strategies for coping with the stress of surviving cancer [36].

*The predominance of death-related stress in cancer*: The prospect of death stands as a primary stressor for individuals with cancer. A significant body of literature has explored the various stress types and coping mechanisms associated with death and dying [37]. Notably, the stress induced by the prognosis following a cancer diagnosis can be more daunting than the approach of death itself. The inherent uncertainty of cancer forces patients to re-evaluate and often postpone their life goals and expectations. Furthermore, due to the unpredictable progression of cancer, conventional coping methods may become ineffective or maladaptive, posing additional challenges for patients navigating this uncertainty [38].

*Multifaceted stressors in cancer patients*: In addition to the primary stress of the disease, cancer patients frequently grapple with fears related to pain and suffering [31]. The physical discomfort and pain originating from the cancer itself are often compounded by the side effects of treatments. Chemotherapy, for instance, is notorious for inducing symptoms like vomiting, fatigue, diarrhea, alopecia (hair loss), and other physical ailments. Similarly, radiation therapy can cause comparable side effects, including significant changes in appearance. Beyond physical symptoms, cancer patients encounter substantial social stressors. Challenges such as the inability to perform routine duties at work, in the community, or at home, and the resultant dependence on others for assistance can precipitate feelings of helplessness and impact self-image, potentially exacerbating feelings of despair. Consequently, cancer patients are likely to experience acute social stressors as a direct consequence of their disease [39].

*Coping strategies in cancer patients*: Van den Borne et al. [40] categorize the major coping strategies employed by cancer patients as follows: seeking information; soliciting support and comfort; assignment (attributing meaning or reason); denial and avoidance of confrontation to preserve self-esteem and ward off negative emotions; active coping strategies; acceptance; and impulsive actions, such as expressions of anger or crying.

Traditionally, religious coping has been perceived primarily as an emotion-focused mechanism, primarily utilized to mitigate negative emotions. However, recent studies indicate that religious coping also encompasses cognitive aspects (such as interpreting illness within the context of divine plans) and behavioral dimensions (including engaging in prayer). This multifaceted nature of religious coping implies that it can be both adaptive (aiding individuals in accepting and adjusting to new circumstances or incurable diseases) and active (enabling individuals to seek comfort and solace through religious practices). Thus, religious coping serves as a vital tool for managing adversities, especially in the context of illness [41].

3. Religious coping strategies

Religious coping can manifest in one of three distinct styles. The 'autonomous style' is characterized by individuals independently tackling problems while maintaining a belief in God, taking active responsibility for their situation. Conversely, the 'letting go style' reflects a stance of deference and patience, where individuals entrust the resolution of their problems entirely to divine intervention, effectively relinquishing personal control. The third, 'collaborative style,' involves a synergistic approach where individuals actively engage in dialogue with God to address their problems, yielding control to a higher power. This collaborative relationship with God is often linked to an enhanced belief in personal ability to handle difficult situations. In contrast, a passive coping style is frequently associated with a diminished sense of personal competence in managing challenges [30].

*Functional versus dysfunctional coping*: Coping mechanisms, in the context of mitigating the adverse psychological impacts of an event, can be categorized as either functional (efficient, adaptive, effective) or dysfunctional (inefficient, maladaptive, ineffective). Functional coping strategies may include accepting the diagnosis and prognosis, maintaining a positive outlook, proactively combating the 'unseen enemy' represented by the disease, adopting an active stance, seeking support from one’s social network, considering all possible disease outcomes, including the less favorable ones, and effectively utilizing available personal resources. On the other hand, dysfunctional coping is exemplified by persistent denial of the illness or diagnosis, harboring anger, withdrawing from social support, concealing the truth, and succumbing to depression. These ineffective coping methods can exacerbate the psychological burden and impede the process of adaptation and healing [42,43].

Pargament et al. [30] argue that exploring religious coping must be theoretically grounded and functionally oriented. They describe five vital religious functions in coping based on various theories:

a. *Meaning*. According to theorists [44], religion plays a vital role in the search for meaning during suffering or difficult life experiences. Religion provides a framework for understanding and interpretation.

b. *Control*. Theorists like Fromm [45] have emphasized the role of religion in seeking control over an event that pushes an individual beyond their resources.

c. *Comfort/spirituality*. According to Freud’s classic theory (1927), religion is designed to reduce the individual's anxieties about living in a world where disaster can strike at any moment.

d. *Intimacy/spirituality*. Sociologists like Durkheim [46] have generally emphasized the role of religion in facilitating social cohesion. Religion is said to be a mechanism for fostering social solidarity.

e. *Life transformation*. Religion can help people make significant life transformations when individuals let go of old values to seek new sources of meaning [29].

Starting from the five dimensions proposed by Pargament [30], several examples of religious/spiritual coping strategies can be indicated (Table 1) [26].

**Table 1:** Examples of religious/spiritual coping strategies.

Religious coping strategies under the five different functions	Positive/negative	Example of coping strategy
1. Meaning-making		
Benevolent religious reappraisal	Positive	Perception of illness as part of a divine plan
Punishing God reappraisal	Negative	Attribution of illness to divine punishment
Demonic reappraisal	Negative	Attribution of illness to evil spiritual forces
Reappraisal of God’s powers	Negative	Doubts regarding the omnipotence of God
2. Regaining control		
Collaborative religious coping	Positive	Joint action plan involving both individual effort and divine guidance
Active religious surrender	Positive	Initial personal effort followed by surrender to divine will
Passive religious deferral	Negative/Mixed	Reliance on divine intervention without personal initiative
Pleading for direct intercession	Negative	Repetitive pleas for miraculous resolution
Self-directing religious coping	Mixed	Attempt to manage distress without recourse to spiritual help
3. Seeking comfort		
Seeking spiritual support	Positive	Engagement in spiritual connection for emotional support
Religious focus	Positive	Use of prayer to divert attention from distress
Religious purification	Positive	Practice of confession as a form of spiritual relief
Spiritual connection	Positive	Intensification of the perceived relationship with God
Spiritual discontent	Negative	Perception of abandonment by God
Marking religious boundaries	Positive	Avoidance of interactions with individuals outside the faith
4. Fostering connection with others/God		
Seeking support from clergy or members	Positive	Engagement with clergy or religious community for guidance
Religious helping	Positive	Use of intercessory prayer on behalf of others
Interpersonal religious discontent	Negative	Friction or disagreement with religious authorities or teachings
5. Life transformation		
Seeking religious direction	Positive	Search for renewed existential purpose through spirituality
Religious conversion	Positive	Embrace of religious conversion as a new direction
Religious forgiving	Positive	Invocation of divine assistance in emotional reconciliation

4. Religious Coping in Romania

*Religiosity in Romania*: According to the World Values Surveys data spanning from 1981 to 2001, Romania ranks among the world's most religious countries. Norris and Inglehart [47] used two key dimensions to measure religiosity: attendance at church services and frequency of prayer. Based on these criteria, Romania is positioned close to highly religious countries such as Ireland, the United States, Uganda, and the Philippines, trailing just behind Iran. Particularly among Orthodox nations, Romania recorded the highest rates of church service attendance and prayer frequency. This assessment was corroborated by the Public Opinion Barometer of November 2005, which also constituted the fifth wave of the World Value Survey. It revealed that a significant majority of the Romanian population (86.5%) identified as Orthodox, followed by 6.1% as Catholics, 2.7% as Protestants, 1.4% as Greek-Catholics, and 2.6% adhering to other denominations. Only a marginal 0.4% of respondents declared no religious affiliation. Notably, 91.5% considered themselves religious. The study underscores that for most Romanians, religious life is deemed necessary or essential, prayer is a habitual practice, and the Church plays a pivotal role in addressing a wide array of life challenges [48].

Given this profound religious inclination, it stands to reason that Romanian patients facing life-threatening events such as cancer would predominantly adopt religious-based psychological adaptation strategies. This hypothesis is supported by Andrada Pârvu's study conducted at the Hematology Clinic in Cluj, between November 2007 and May 2009. The findings indicate a significant reliance on religious coping mechanisms among Romanian cancer patients [49].

*Methodological approach to religious coping*: The study commenced with an application of Pargament's classification, which delineates three primary mechanisms of religious coping. These include 'collaborative' coping, where control over the situation is shared between the individual and the divine; 'self-management,' where the individual assumes direct control, acknowledging but not solely relying on divine intervention; and 'postponement,' characterized by attributing full control to divine forces. The first two methods are generally regarded as more adequate coping strategies.

*Incorporation of Kubler-Ross's stages*: Employing the Kubler-Ross model, the study quantitatively assessed emotional responses in cancer patients. The findings indicated that denial was present in 71% of the cases, depression in 70%, anger in 15%, and acceptance in 95%. These percentages reflect the diverse emotional landscapes navigated by patients during their illness.

*Hierarchy of psychological resources*: An evaluation of psychological resources used by patients revealed that family support ranked highest. This was followed by reliance on psychologists (90%), religious faith (89%), and medical doctors (82%). Notably, support from friends was less prevalent, ranking lowest at 61%.

The study also documented cases among terminal patients, highlighting the influence of pastoral care. Priests in support groups were instrumental in guiding patients towards reinterpreting their illness as a catalyst for returning to fundamental values such as religion and family, and in rediscovering spiritual values. This guidance facilitated patients in accepting their suffering while nurturing or augmenting their faith, transforming their suffering into a spiritually valuable experience, and maintaining a proactive attitude.

*Ineffective religious coping mechanisms*: The study identified several religious coping mechanisms deemed ineffective by most patients. These included a passive attitude, loss of faith, doubts regarding the existence of divinity, blaming divine entities for the onset of their illness, and interpreting the disease solely as a form of divine punishment [50,51]. Such approaches are not recommended for clergy providing spiritual care in hospital settings. Research indicates that negative religious coping is associated with increased psychological distress and a higher risk of mortality [52].

During the study, religion was noted to play several positive roles in effective coping. These include the reduction of anxiety and stress, enhancement of overall well-being, improvement of mental and physical health, provision of comfort to the soul, aiding in the search for meaning in suffering, and encouraging the adoption of healthy lifestyles [53].

The Romanian Orthodox Church advocates for a comprehensive approach in treating patients with malignant diseases. This approach prioritizes spiritual measures, including the administration of Holy Sacraments, spiritual counseling, prayer, and moral support. Concurrently, it emphasizes the importance of medical interventions, such as standard treatments, maintaining proper hygiene, and palliative care, to alleviate the pain and discomfort associated with the disease [54].

*Role of prayer in coping*: Prayer has been recognized as a significant strategy for fostering hope among cancer patients, as evidenced in various studies [55]. Taylor et al.'s investigation [28] provides detailed insights into how patients utilize prayer to mitigate their physical, emotional, and spiritual suffering caused by cancer. Different stages of the disease elicit varied prayer themes: for instance, prayers during the diagnostic phase often seek guidance for treatment decisions, while those in the terminal phase may focus on requests for a peaceful death, comfort for loved ones, and seeking forgiveness. Patients reported that prayer primarily offered emotional relief, with fewer acknowledgments of physical betterment or healing. The study also identified factors that facilitate prayer, as well as obstacles, such as medication-induced cognitive impairment, distractions, and interruptions during hospital stays.

Within the Christian Orthodox faith, illness is often interpreted as a divine summons towards piety, humility, and the pursuit of salvation, paralleling the quest for physical healing. Suffering induced by disease is viewed as an opportunity for reconciliation with one's past and with God, easing of the conscience, and guiding the individual towards confession. This perspective posits that a devout Christian should depart this world reconciled with God and fellow humans, with the Holy Communion serving as a testament to their forthcoming personal encounter with God and the promise of eternal communion with the divine [56].

Limitations, recommendations, and future directions

Despite offering valuable insights into the role of religious coping strategies, particularly within the Christian Orthodox context, several limitations should be acknowledged. First, the review is based primarily on qualitative synthesis and data from different reported studies, which may vary in methodological rigor and cultural context. Thus, it could introduce potential heterogeneity and limits the generalizability of the findings. Second, although Romania serves as a representative case for Eastern Orthodox religiosity, the findings may not be applicable to non-Orthodox cancer patient populations, nor to Orthodox populations outside Eastern Europe. Moreover, due to the heterogeneity in study designs, measures of religiosity, and conceptual outcomes across the included studies, a formal meta-analysis was not feasible. Therefore, we conducted a qualitative thematic synthesis.

Given these limitations, future studies should aim to develop validated instruments to quantitatively assess religious coping in cancer patients, including in the Eastern Orthodox context. Also, studies are recommended to explore the evolving role of spirituality throughout the cancer trajectory, from diagnosis to outcomes and end-of-life care. Furthermore, interdisciplinary collaborations between oncologists, psychologists, and palliative care specialists are essential to better integrate spiritual care into holistic cancer management.

## Conclusions

In summary, the trajectory of research within psychosocial oncology over recent decades marks a significant shift in the understanding and appreciation of religion and spirituality in coping with diseases. Historically understudied and treated peripherally, these dimensions have now emerged as crucial factors in the appraisal and coping processes for individuals facing life-threatening illnesses. Contemporary studies have illuminated the profound correlation between religious factors and patients' adaptation to diseases, particularly cancer. They underscore that religiosity is not merely a peripheral aspect but is intrinsically linked to fostering hope, meaning, and peace in patients. The frequent reliance on prayer and a profound trust in God, as reported by many patients, underscores the efficacy of these spiritual strategies in mitigating the stress associated with cancer survival. This paradigm shift underscores the essential role of religious and spiritual resources, especially in contexts of severe stress and existential threat. Recognizing and integrating these spiritual dimensions into holistic care models can significantly enhance support mechanisms for patients grappling with life-threatening diseases, paving the way for more comprehensive and empathetic healthcare approaches.
